# Complete sequence and detailed analysis of the first indigenous plasmid from *Xanthomonas oryzae* pv. *oryzicola*

**DOI:** 10.1186/s12866-015-0562-x

**Published:** 2015-10-24

**Authors:** Xiang-Na Niu, Zhi-Qiong Wei, Hai-Fan Zou, Gui-Gang Xie, Feng Wu, Kang-Jia Li, Wei Jiang, Ji-Liang Tang, Yong-Qiang He

**Affiliations:** State Key Laboratory for Conservation and Utilization of Subtropical Agro-bioresources, The Key Laboratory of Ministry of Education for Microbial and Plant Genetic Engineering, and College of Life Science and Technology, Guangxi University, 100 Daxue Road, Nanning, 530004 China

**Keywords:** *Xanthomonas oryzae* pv. *oryzicola*, Indigenous plasmid, Self-transmissible, Tn*3*-like transposon, Heavy metal tolerance

## Abstract

**Background:**

Bacterial plasmids have a major impact on metabolic function and adaptation of their hosts. An indigenous plasmid was identified in a Chinese isolate (GX01) of the invasive phytopathogen *Xanthomonas oryzae* pv. *oryzicola* (*Xoc*), the causal agent of rice bacterial leaf streak (BLS). To elucidate the biological functions of the plasmid, we have sequenced and comprehensively annotated the plasmid.

**Methods:**

The plasmid DNA was extracted from *Xoc* strain GX01 by alkaline lysis and digested with restriction enzymes. The cloned and subcloned DNA fragments in pUC19 were sequenced by Sanger sequencing. Sequences were assembled by using Sequencher software. Gaps were closed by primer walking and sequencing, and multi-PCRs were conducted through the whole plasmid sequence for verification. BLAST, phylogenetic analysis and dinucleotide calculation were performed for gene annotation and DNA structure analysis. Transformation, transconjugation and stress tolerance tests were carried out for plasmid function assays.

**Results:**

The indigenous plasmid from *Xoc* strain GX01, designated pXOCgx01, is 53,206-bp long and has been annotated to possess 64 open reading frames (ORFs), including genes encoding type IV secretion system, heavy metal exporter, plasmid stability factors, and DNA mobile factors, i.e., the Tn*3*-like transposon. Bioinformatics analysis showed that pXOCgx01 has a mosaic structure containing different genome contexts with distinct genomic heterogeneities. Phylogenetic analysis indicated that the closest relative of pXOCgx01 is pXAC64 from *Xanthomonas axonopodis* pv. *citri* str. 306. It was estimated that there are four copies of pXOCgx01 per cell of *Xoc* GX01 by PCR assay and the calculation of whole genome shotgun sequencing data. We demonstrate that pXOCgx01 is a self-transmissible plasmid and can replicate in some *Xanthomonas* spp. strains, but not in *Escherichia coli* DH5α. It could significantly enhance the tolerance of *Xanthomonas oryzae* pv. *oryzae* PXO99^A^ to the stresses of heavy metal ions. The plasmid survey indicated that nine out of 257 *Xoc* Chinese isolates contain plasmids.

**Conclusions:**

pXOCgx01 is the first report of indigenous plasmid from *Xanthomonas oryzae* pv. *oryzicola*, and the first completely sequenced plasmid from *Xanthomonas oryzae* species. It is a self-transmissible plasmid and has a mosaic structure, containing genes for macromolecule secretion, heavy metal exportation, and DNA mobile factors, especially the Tn*3*-like transposon which may provide transposition function for mobile insertion cassette and play a major role in the spread of pathogenicity determinants. The results will be helpful to elucidate the biological significance of this cryptic plasmid and the adaptive evolution of *Xoc*.

**Electronic supplementary material:**

The online version of this article (doi:10.1186/s12866-015-0562-x) contains supplementary material, which is available to authorized users.

## Background

*Xanthomonas oryzae* pathovar *oryzicola* (here after, *Xoc*), one of the major pathogenic bacteria in rice, can cause rice bacterial leaf streak (BLS) resulting in significant reduction in yield and economic losses of rice production [1]. The pathogen invades rice mainly through leaf stomata or wounds and colonizes intercellular spaces in the mesophyll, resulting in water-soaked interveinal lesions that can develop into translucent streaks [1]. Rice resistance to BLS was found to be a quantitative trait and no major resistance genes were identified in rice up to now [2]. BLS is principally controlled by crop spraying with copper compounds and a few agricultural antibiotics. This seldom results in ideal control and often gives rise to environmental concerns. As a result, BLS is gradually becoming one of the major limiting factors to rice production in the tropical and sub-tropical areas in Asia and Africa [1, 3, 4].

Plasmids are extrachromosomal autonomously replicating DNA molecules, which often carry genes that may benefit the survival of the host organism, such as antibiotic resistance, heavy metal tolerance, and toxin production [5]. They are also known as a type of mobile genetic element which plays very important roles in horizontal gene transfer and gene exchange in nature [6]. Substantial numbers of animal and plant pathogenic bacteria were found harboring plasmids which effected their adaptation, pathogenicity and evolution [5, 7].

Plasmids have been reported from many xanthomonads. *X. albilineans*, *X. arboricola* pv. *pruni*, *X. axonopodis* pathovars *cyamopsidis*, *dieffenbachiae, glycines*, *manihotis*, *phaseoli*, *vignicola*, and *vitians*, and *X. citri* pv. *citri, X. campestris* pathovars *campetris*, *malvacearum* and *vesicatoria,* and *X. hortorum* pathovars *hederae* and *pelargonii,* and *X. fuscans* subsp. *fuscans* and *X. oryzae* pv. *oryzae* possess at least one type of plasmid in each bacterium [8–17]. The complete DNA sequences of some plasmids have been published from *X. albilineans*, *X. arboricola* pv. *pruni*, *X. axonopodis* pv. *citri*, *X. axonopodis* pv. *glycines*, *X. fuscans* subsp. *fuscans*, *X. campestris* pv. *campestris* and *X. campestris* pv. *vesicatoria* [12–19]. Plasmids from *Xanthomonas* are significantly diverse in size and gene composition. Some of them carry genes encoding macromolecule secretion systems, effectors, heavy metal exporters, plasmid stability factors, and DNA mobile elements. A range of plasmid-mediated phenotypes, including virulence, toxin and hormone production, and resistance to bactericides, have been reported in many other phytopathogenic bacteria [11]. However, the plasmid biology of *Xanthomonas* is still not well understood [11, 12].

To date, hundreds of *Xoc* strains have been isolated and identified from Asia and Africa [20–23], and complete genomes of two *Xoc* strains BLS256 from the Philippines [24] and CFBP7342 from Burkina Faso (GenBank Accession: CP007221) have been determined. However, there are no reports about plasmids in any *Xoc* strains, or a complete plasmid DNA sequence from *X. oryzae* species.

In our previous study, a rifampicin-resistance spontaneous mutant, named GX01 [25], was selected from the wild type strain LT4, which was isolated from the rice leaf with typical BLS symptoms in Liantang Town of Hezhou City of Guangxi, in the central area of South China rice growing regions, where a specific population of the wild rice (*Oryza rufipogon*) is thought to be the most recent ancestor of *Oryza sativa japonica* [26]. A cryptic plasmid was found by chance in this high virulent strain GX01. To our knowledge, this is the first report about indigenous plasmids in *X. oryzae* pv. *oryzicola*. In this study, we sequenced and comprehensively annotated this conjugative plasmid.

## Methods

### Bacterial strains, plasmids, and general culture conditions

Bacterial strains and plasmids used in this study are listed in Table 1.

**Table 1:** Bacterial strains and plasmids

Strains or plasmids	Relevant traits^a^	Source or reference
Strains		
*Xanthomonas* spp.		
*X. oryzae* pv. *oryzicola* GX01	Harboring plasmid pXOCgx01; Rif^r^	This study
*X. oryzae* pv. *oryzicola* isolates	Isolates from China; some harboring plasmids	
*X. campestris* pv. *campestris* 8004	plasmidless; Rif^r^	[57]
*X. oryzae* pv. *oryzae* PXO99^A^	plasmidless; Rif^r^ (a rifampicin resistant mutant selected in our lab)	[58]
*Xoo* PXO99^A^/pXOCgx01::Tn5	PXO99^A^ harboring plasmid pXOCgx01 insertion with Tn5; Rif^r^, Kan^r^	This study
*Enterobacteriaceae*		
EC100D *pir* ^+^	F^−^, Ф80d/*lac*ZM15, *lac*X74, *rec*A1, *end*A1, *gal*U, *mcr*A, *gal*K, λ^−^, *rps*L, *ara*D139, *nup*G, *pir*+	Eppicentre Biotechnologies
DH5α	F^−^, Ф80d/*lac*ZΔM15, *deo*R, *end*A1, *hsd*R17, *pho*A, *sup*E44, λ^−^, *thi*-1, *gyr*A96, *rel*A1	Our library
Plasmid		
pUC19	Cloing vector; Amp^r^	Our library

^a^Rif^r^, Kan^r^ and Amp^r^ indicated resistance to rifampin, kanamycin and ampicillin, respectively

*Xoc* strains and *Xoo* PXO99^A^ were cultured in Nutrient Broth (NB) medium (per liter: 5.0 g hipolypeptone, 1.0 g yeast extract, 3.0 g beef extract, 10.0 g sucrose, pH 7.0) at 28 °C and *Xcc* 8004 was cultured in NYGB medium (per liter: 5.0 g peptone, 3.0 g yeast extract and 20.0 g glycerol) [27] at 28 °C. *Escherichia coli* strain DH5α and EC100D™ *pir* + were cultured in Luria–Bertani (LB) broth (per liter: trytone 10.0 g, yeast extract 5.0 g, NaCl 10.0 g) at 37 °C. When required, media were supplemented with antibiotics as follows: 50 μg/mL rifampicin (Rif), 25 μg/mL kanamycin (Kan) or 100 μg/mL ampicillin (Amp).

### Plasmid isolation and identification

Plasmid DNA from *E. coli* cells and *Xoc*/*Xoo*/*Xcc* strains were extracted by the alkaline lysis method as described by O’Sullivan and Klaenhammer [28] with some modifications. To estimate the size and profile polymorphisms of plasmids from different *Xoc* strains, digestion reactions with different restriction endonucleases were done after plasmid harvest, and all the samples were checked by 0.8 % agarose gel electrophoresis.

### Plasmid DNA sequencing

Good quality plasmid DNA fragments of pXOCgx01 were isolated and selected by digestion with *Bam*HI, *Pst*I, *Sph*I and *Eco*RI, respectively, and were cloned into vector pUC19 with corresponding sites. 15 pUC_*Bam*HI_ subclones, 22 pUC_*Eco*RI_ subclones, 39 pUC_*Pst*I_ subclones and 48 pUC_*Sph*I_ subclones were gotten, and sequenced by Sanger sequencing using vector specific M13 primer pair. Sequences were assembled by using Sequencher software. Meanwhile, whole-genome shotgun sequencing of *Xoc* strain GX01 was performed using the Illumina platform, and sequences were assembled by using SOAPdenovo Packages. Gaps were closed by primer walking and sequencing, and at last multi-PCR were done through the whole plasmid sequence for verification.

### Annotation and bioinformatics analysis

Open reading frames (ORFs) containing more than 30 amino acid residues were predicted using Glimmer V3.02 [29] and GeneMarkS V4.30 [30], and verified by manually analysis. Potential protein-coding sequences were subsequently analyzed manually using BLAST suite of programs, including BLASTN, BLASTP, BLASTX, clusters of orthologous groups (COG) and conserved domain database (CDD). The protein motifs and domains of all ORFs were characterized based on intensive searches against public databases using Interproscan tools. tRNA genes were identified by using tRNAscan-SE. GC skew analysis and the circular-genome-map drawing were performed using BRIG software [31].

Phylogenetic analyses of gene clusters were performed with BLASTN search and multiple alignments were developed with MEGA 6 [32] and PHYLIP. The phylogenetic tree of the whole sequences of plasmid pXOCgx01 was drew by using the online tool EvolView described by Zhang [33].

The method of the Delta similarity (δ^*^) calculation is based on the method described by Karlin method [34]:

$$ {\delta}^{*}\left(f,g\right)=\raisebox{1ex}{$1$}\!\left/ \!\raisebox{-1ex}{$16$}\right.{\displaystyle \sum_{XY}}\left|{\rho}_{XY}^{*}(f)-{\rho}_{XY}^{*}(g)\right| $$

Here f and g is the FASTA sequence of the two compared genome DNA sequence and XY is the combination of the 4 nucleotide base ATG, function ρ is the odds ratio of the two nucleotide base X and Y:

$$ {\rho}_{XY}=\frac{f_{XY}}{f_X{f}_Y} $$

Here function *f*_*x*_ is the frequency of the nucleotide base X occurs in the fasta sequence f or g, and function *f*_*XY*_ is the frequency of the dinucleotide XY in the sequence under study.

### PCR assays

For screening for the presence of plasmid pXOCgx01, single clones were selected and transferred to a new NA medium plate, and pXOCgx01-specific primers targeting genes involved in pXOCgx01 T4SS genes, resolvase genes and transposase genes were designed using the program Vector NTI V11.5. Single bacterial colony was scraped off an NA culture plate with a sterile toothpick, and was resuspended in 4 μL sterile deionized water in an Eppendorf PCR tube as a DNA template. Amplifications were carried out in a final volume of 20 μL consisted of 12.4 μL sterile deionized water, 2 μL 10 × *Taq*-DNA polymerase buffer, forward and reverse primers at 0.25 μM each, dNTPs at 0.25 mM, 1 units *Taq*-DNA polymerase and 4 μL DNA template sample prepared above. The PCR products were resolved on 1.2 % agarose gels at 100 volts for about 35 min, stained with nucleic acid gel stain GelRed and photographed under UV light (BIO-RAD UNIVERSAL HOOD II).

### Obtainment of the plasmid selection marker

As pXOCgx01 has no antibiotic selection markers used in our lab, a kanamycin resistance marker was inserted using EZ-Tn5™ < R6Kγori/KAN-2 > Insertion Kit (Cat. No. EZI011RK) according to the manufacturer’s instructions in vitro. The randomly inserted plasmids were transferred into EC100D *pir*^+^ by electroporation and clones harboring pXOCgx01::Tn5 were selected on LA amended with kanamycin. An EZ-Tn5™ < R6Kγori/KAN-2 > transposon-specific PCR-checking was established using primers Tn5-R6K-F/R (Additional file 1). Plasmids with Tn5 cassette from EC100D *pir*^+^ and the plasmid from the wild-type strain *Xoc* GX01 were extracted and digested with *Bam*HI, followed by the contrastive analysis by 0.8 % agarose gel electrophoresis to make sure no fragments were trimmed in these transformants. The insertion site of the Tn5 cassette in pXOCgx01::Tn5 was determined through “Rescue Cloning” method. Briefly, plasmid DNA was extracted from EC100D *pir*^+^, and digested with one restriction endonuclease which no corresponding site exists in EZ-Tn5™ < R6Kγori/KAN-2 > Transposon like *Eco*RI. Then the digested DNA was purified and circularized by self-ligation. The mini-Tn5 cassette with the flanking DNA of the insertion site was transferred into EC100D *pir*^+^ by electroporation and clones were selected on LA amended with kanamycin. Positive clones were selected using primers Tn5-R6K-F/R as above and the mini-Tn5 cassette DNA was extracted by the alkaline lysis method and digested with the same enzyme as above and followed by electrophoresis analysis on 0.8 % agarose gel. Clones with different profiles which might be different insertion-site clones were chosen for sequencing using the Tn5 transposon-specific primers Tn5KAN2-F/Tn5R6KAN2-R (Additional file 1). Sequence analysis showed that one Tn5 cassette inserted in plasmid pXOCgx01 at the spacer region between XOCp0043 and XOCp0044. This clone was designated as pXOCgx01::Tn5.

### Conjugal transfer of pXOCgx01

EC100D *pir*^+^/pXOCgx01::Tn5 was used as a donor to track conjugal transfer of pXOCgx01 to *Xoo* PXO99^A^. Donor and recipient strains were harvested in midlogarithmic phase and washed with 0.9 % saline solution and NB medium, mixed, and placed on NA medium plates without any antibiotics. After incubation at 30 °C for 24 h, cells were picked up to sterile Eppendorf tubes and suspended with 300 μL NB medium. The mixed cells were spread on NA medium amended with rifampin and kanamycin and incubated at 28 °C for 4 to 5 days. Putative transconjugants were examined with Tn5 cassette-specific primers Tn5-R6K-F/R, pXOCgx01-specific primers pXOC-virF/R, pXOC-res-F/R, pXOC-tra-F/R (see Additional file 1). Plasmid DNA was isolated from the putative transconjugants by the modified alkaline lysis method as above and digested with *Bam*HI. Fragment profiles were examined with agarose gel electrophoresis.

### Curing of plasmid pXOCgx01 from strain GX01

Kinds of approaches were adopted to cure plasmid pXOCgx01 from strain GX01. *Xoc* GX01 was grown at an elevated temperature (37 °C) in liquid NB medium, passaging every 24 h for a week. Cells were then diluted in NB medium and plated onto NA plates with rifampin. Single colonies (*n* = 500) were subsequently screened for the sequence of plasmid pXOCgx01 with the PCR assay as described above (use primers pXOC-virF/R, pXOC-res-F/R and pXOC-tra-F/R, see Additional file 1). And 10 colonies were selected to isolate plasmid DNA and be digested with *Bam*HI for fragment analysis. Besides, GX01 was grown in NB medium with SDS (0.008 to 0.015 % final concentrations) at 28 °C, passage every 24 h for a week, and plasmid pXOCgx01 was checked as above. SDS and elevated temperature crossed method was also attempted. Other ways have been also conducted, such as acridine orange (1 μg/mL to 20 μg/mL final concentration) stress tests, and *Xoc* GX01 electrocompetent cells electric pulse tests (2-mm cuvette with a Bio-Rad Gene Pulser Xcell at 2000 to 3000 V for Time constant protocol, capacitance and resistivity were variable; 1000 to 2000 V for Square Wave Protocol, pulse length: 5 ms, number of pulses: 2, pulse interval: 5 s).

Some broad-host-range plasmids such as pLAFR6, pPH1JI, pBBad18K, pUFR034 and pBBR1MSC-5 were introduced into *Xoc* GX01 by electrotransformation in order to find an incompatibility plasmid and cure pXOCgx01 from *Xoc* GX01. The putative origin (coordinates 48,376–53,206 joined with coordinates 1–7175) was cyclized with the EZ-Tn5™ < R6Kγori/KAN-2 > transposon and introduced into *Xoc* GX01 by electrotransformation. Transformants were selected from NA plates with relative antibiotics, and positive clones were passaged every 24 h in 5 mL NB medium with relative antibiotics for a week. At each passage, aliquots of each culture were diluted and spread on NA plates with relative antibiotics. Single clones were screened by colony PCR described above to check the presence of the plasmid pXOCgx01.

### Nucleotide sequence accession number

The complete sequence of plasmid pXOCgx01 has been deposited in GenBank under accession number KR071788.

## Results

### Identification and nucleotide sequencing of plasmid from *Xoc* strain GX01

**Fig. 1:**
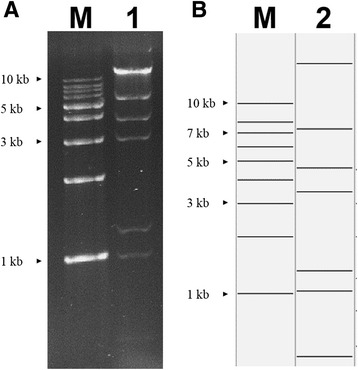
Restriction fragment patterns digested with *Bam*HI of the isolated extrachromosomal DNA molecule from *Xoc* GX01. **a** the actual restriction enzyme electrophoresis (*lane 1*); **b** the simulated enzyme map (*lane 2*) by using Vector NTI software. M denotes DNA ladder marker (TianGen, 1 kb DNA Ladder)

An extrachromosomal DNA molecule was found in *Xoc* GX01 by comparing restriction fragment patterns of *Xoc* GX01 DNA samples extracted with different approaches. To determine its entire DNA sequence, the extrachromosomal DNA was extracted by alkaline lysis and digested with *Bam*HI, *Eco*RI, *Pst*I and *Sph*I respectively, followed by being subcloned into pUC19 vector for sequencing. Meanwhile, whole-genome shotgun sequencing of *Xoc* GX01 was performed by using the Solexa sequencing method. By assembling the subclones’ sequencing and the genome sequencing data, followed by gap filling and PCR checking, a 53,206-bp circular DNA molecule was generated. The actual electrophoresis of the isolated extrachromosomal DNA molecule after digestion with *Bam*HI was in accordance with the simulated result of the assembled circular molecule by using Vector NTI software (Fig. 1). Some plasmid-related protein genes, such as those for plasmid stability were predicted and the potential *oriV* and *oriT* were identified by sequence similarity searching pA506 and pXAC64 from *Pseudomonas fluorescens* A506 [35] and *X. citri* pv. *citri* 306 [36] respectively. These results indicated that the extrachromosomal DNA molecule from *Xoc* GX01 was a cryptic plasmid. And from the sequencing results and the plasmid profiles, we confirmed that there was only one type of plasmid in strain GX01, thus we designated this extrachromosomal DNA molecule as plasmid pXOCgx01. The copy number of pXOCgx01 was estimated to be four per cell, based on the Solexa sequencing data calculation, by dividing the coverage depth of the chromosome-related reads by that of the plasmid-related reads.

### General overview of the plasmid pXOCgx01

**Fig. 2:**
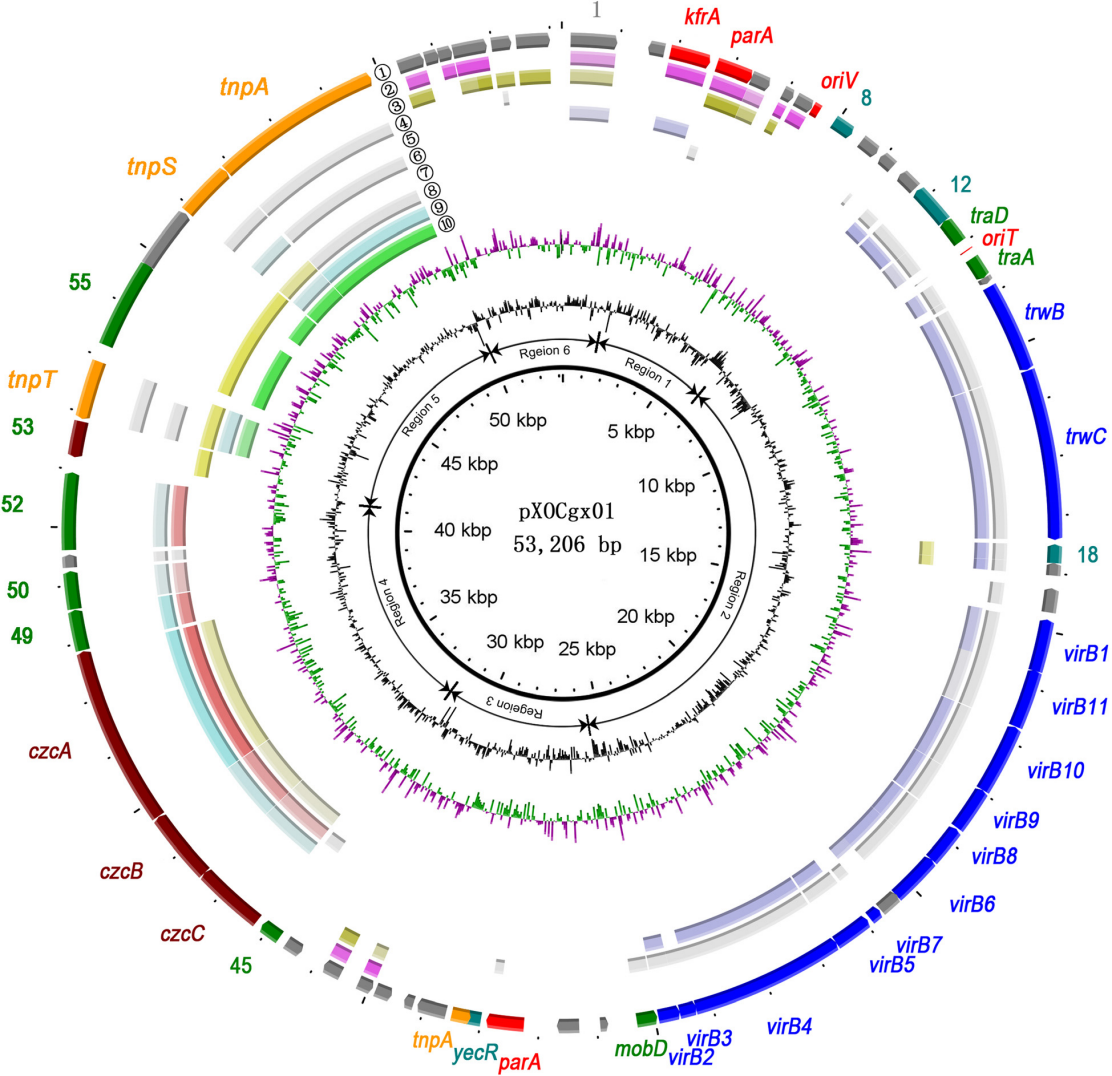
Circular map of plasmid pXOCgx01. The outer circle shows predicted coding sequences. Different colors represent different putative functions: gray, (conserved) hypothetical protein; red, intergenic regions with putative functions as the origin of transfer (*oriT*), origin of vegetative replication (*oriV*), and plasmid replication relative proteins as *kfrA* and *parA* proteins; green, transmembrane proteins and conjugal transfer proteins; blue, T4SS locus; maroon, metal resistance relative proteins, like *czcABC* locus and metal-binding proteins; teal, other proteins as CcgAII protein, putative PemK-like protein, plasmid stable inheritance protein, *yecR*. Circles 2 through 10 depict nine other plasmid or chromosome genomes owning conserved regions in pXOCgx01 as determined by Blastp (cutoff of 1e-5). Second circle, plasmid I from *Xanthomonas campestris* pv. *campestris* B1459; third circle, plasmid pla from *Xanthomonas fuscans* subsp. *fuscans* str. 4834-R; fourth circle, pXAC64 from *Xanthomonas axonopodis* pv. *citri* str. 306; fifth circle, pXCV38 from *Xanthomonas campestris* pv. *vesicatoria* str. 85–10; sixth circle, *Pseudomonas aeruginosa* UCBPP-PA14; seventh circle, *Stenotrophomonas. maltophilia* K279a; eighth circle, *Pseudoxanthomonas spadix* BD-a59; ninth circle, pXCV183 from *Xanthomonas campestris* pv. *vesicatoria* str. 85–10; tenth circle, *Xanthomonas oryzae* pv. *oryzae* PXO99^A^. Within each circle of the nine, the darkest color indicates nucleotide identity exceeding 70 % whereas the lightest color represents identity exceeding 40 %. Eleventh circle, G + C content. Twelfth circle, GC skew. The circular plasmid diagram was generated using BRIG

The total length of the indigenous plasmid pXOCgx01 is 53,206 bp, with an average G + C content 61.25 %, lower than that of the *Xoc* GX01 chromosome (64.08 %) and that of other xanthomonads genomes [7, 17, 24]. Of all the 64 open reading frames (ORFs), 28 were predicted to be transcribed from the leading replication strand (Fig. 2 and Additional file 2), and 37 were functionally assigned while 23 were predicted to encode conserved hypothetical proteins by homologous sequence search and domain characterization, whereas four ORFs could only be annotated as hypothetical proteins showing plasmid specificity. The average length of ORFs is 713 bp, similar to that of plasmids in other *Xanthomonas* [7]. One tRNA (tRNA-Ile) gene, with a length of 73 bp (coordinates 41,134–41,206), was identified using tRNAscan-SE. The *virB* and *czcCBA* loci were identified as well as plasmid replication and transportation protein genes. Unlike plasmids in other xanthomonads, no antibiotic resistance, copper resistance or type III effector related genes were identified in pXOCgx01 [7, 11, 12, 37]. Phylogenetic analysis of pXOCgx01 with the plasmids from GenBank reveals that the global similarity of pXOCgx01 with the vast majority plasmids is quite low, with less than 30 % sequence coverage. We found that only the plasmid pXAC64 from *Xac* 306 shares an identity of 96 % with 31 % sequence coverage against pXOCgx01 (Additional file 3).

### The mosaic structure of pXOCgx01 revealed by comparative and phylogenetic analyses

Comparative analyses and BLAST results of pXOCgx01 to GenBank showed that different parts of the plasmid have distinctly different similarities to other genomes (Additional file 4). Six DNA regions with obviously different genomic heterogeneities have been grouped based on G + C content, dinucleotide bias (δ*-differences) and BLAST results (Tables 2 and 3), outlining the mosaic structure of pXOCgx01. Of the six clusters, four have syntenic regions in the genomes of other organisms, but the other two have not. The longest cluster is 19,095 bp in length, which has a high identity to the *virB* locus on the plasmid pXAC64 of *Xac* 306 [15]. The second longest cluster is the *czcABC* locus containing 8 ORFs, which may represent one of the ancient DNA segments for heavy metal exclusion, identified basing on the phylogenetic analysis from gene-order data [38]. About one fourth of the plasmid genes have few significant homologs in GenBank based on the BLASTN search, especially the (conserved) hypothetical protein genes. The mosaic structure indicated that the generation of pXOCgx01 may be an event of multiple DNA rearrangements by horizontal gene transfers.

**Table 2:** Plasmid DNA context features of pXOCgx01

Features	Region I	Region II	Region III	Region IV	Region V	Region VI
Coordinates	1492–6218	6219–25,313	25,314–31,747	31,748–41,053	41,054–49,841	49,842–1491
Length (bp)	4727	19,095	6434	9306	8788	4856
GC (%)	60.93	59.54	59.61	63.79	61.91	64.44
δ*-differences	88.58	59.23	66.91	65.39	86.15	88.51
Functions	KrfA, ParA	*oriT*, T4SS	hypothetical	CzcCBA	Tn*5044*	hypothetical

δ*-differences (dinucleotide bias) indicate the cumulative dinucleotide differences between each region and the segment from *dnaA* to *gyrB* of chromosome of *Xoc* GX01

**Table 3:** Dinucleotide bias (δ*-differences) between DNA regions of pXOCgx01

Regions	Region I	Region II	Region III	Region IV	Region V	Region VI
Region I	0	99.25	76.07	103.63	132.99	70.40
Region II		0	80.54	84.07	94.82	95.75
Region III			0	79.73	74.62	72.29
Region IV				0	55.27	100.80
Region V					0	111.94
Region VI						0

δ*-differences indicate the cumulative dinucleotide differences between each region

**Fig. 3:**
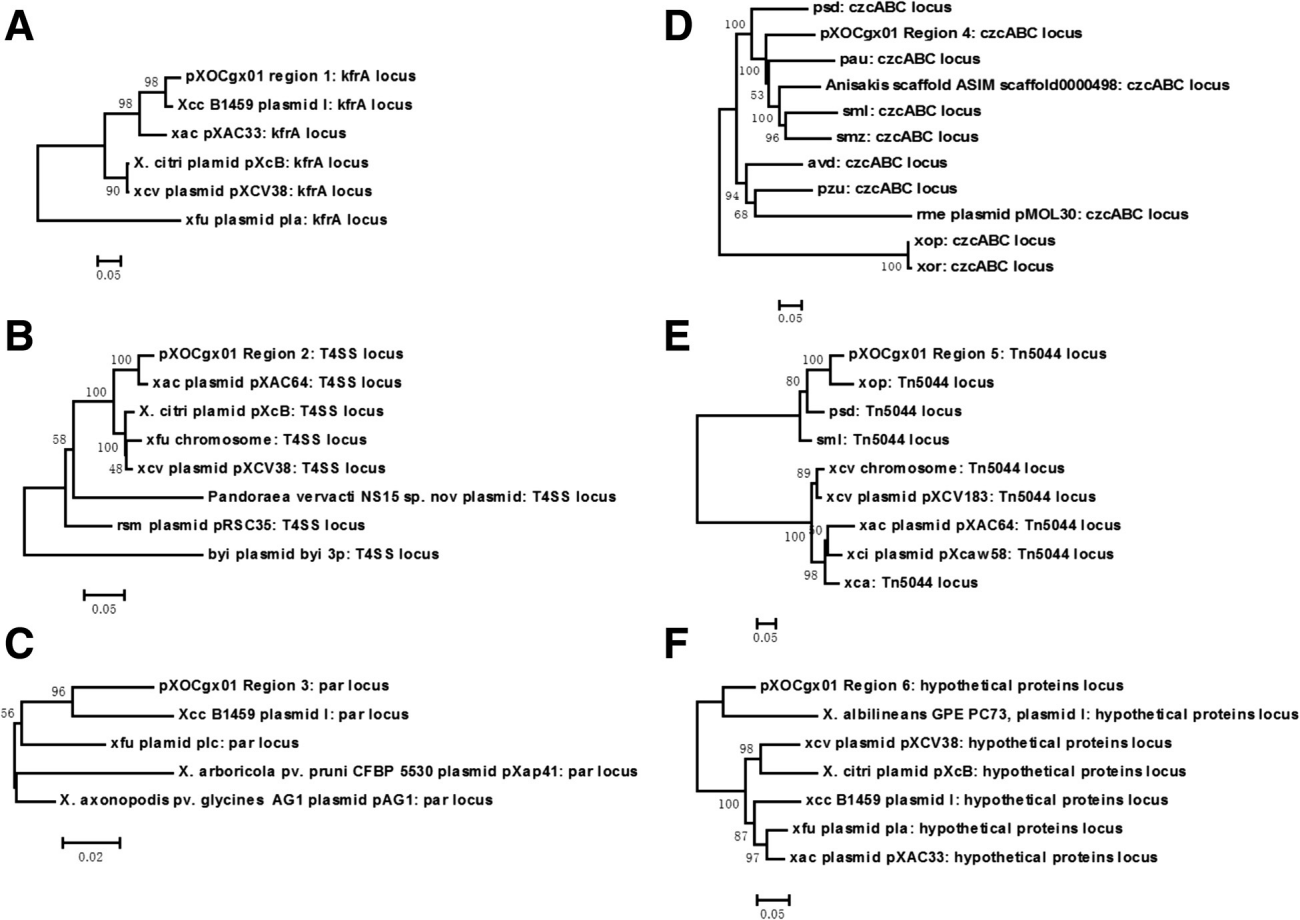
Unrooted phylogenetic tree (Neighbor-Joining) for six conserved regions in pXOCgx01. **a**. Region I; **b**. Region II; **c**. Region III; **d**. Region IV; **e**. Region V; **f**. Region VI. DNA sequences of other genomes were abstracted from Genbank. The specific locations are summarized in Additional file 5. Abbreviation of genome names: *Xcc* B1459 plasmid I: *Xanthomonas campestris* pv. *campestris* B1459 plasmid I; *xfu* plasmid pla: *Xanthomonas fuscans* subsp. *fuscans* str. 4834-R plasmid pla; *xac* plasmid pXAC33: *Xanthomonas axonopodis pv. citri* str. 306 plasmid pXAC33; *xac* plasmid pXAC64: *Xanthomonas axonopodis pv. citri* str. 306 plasmid pXAC64; *xcv* plasmid pXCV38: *Xanthomonas campestris* pv. *vesicatoria* str. 85–10 plasmid pXCV38; *byi* plasmid byi_3p: *Burkholderia* sp. YI23 plasmid byi_3p; *rsm* plasmid pRSC35: *Ralstonia solanacearum* CMR15 plasmid pRSC35; *X. citri* plasmid pXcB: *Xanthomonas citri* plasmid pXcB; *avd*: *Azotobacter vinelandii* CA6; *pzu*: *Phenylobacterium zucineum* HLK1; *pau*: *Pseudomonas aeruginosa* UCBPP-PA14; *psd*: *Pseudoxanthomonas spadix* BD-a59; *smz*: *Stenotrophomonas maltophilia* D457; *sml*: *Stenotrophomonas maltophilia* K279a; *xop*: *Xanthomonas oryzae* pv. *oryzae* PXO99^A^; *xcv* plasmid pXCV183: *Xanthomonas campestris* pv. *vesicatoria* plasmid pXCV183; *xca*: *Xanthomonas campestris* pv. *campestris* complete genome, strain B100; *xci* plasmid pXcaw58: *Xanthomonas citri* subsp. *citri* Aw12879 plasmid pXcaw58

To obtain some insight into the evolution, unrooted phylogenetic trees were constructed from nucleic acid sequences of each region (Fig. 3, Additional file 5). From these phylogenetic trees, we could clearly see that different loci of pXOCgx01 derived from different species. For instance, the closest evolutionary relative with the *kfrA* locus was that of *X*cc B1459 plasmid I; and the T4SS locus had the closest relationship with that of pXAC64 of *Xac* 306, while the Tn*5044* locus with that of *X*oo PXO99^A^. These results were in accordance with dinucleotide bias analysis above, supporting the mosaic structure of pXOCgx01.

### Putative genes for type IV secretion system and conjugation

Gene annotation revealed that the *virB* locus of pXOCgx01 encodes almost the complete set of proteins for the Type IV secretion system (T4SS) [39], precisely the subgroup A of T4SS (T4ASS) which was identified on the Ti plasmid in *Agrobacterium tumefaciens* [40]. The comparative analysis showed that the distribution of *virB* loci differed significantly in xanthomonads (Additional file 6). By comparing with the T4ASS from *A. tumefaciens*, almost none of the xanthomonads have a typical *virB7* homolog in their *virB* loci. Recently, Souza et al. [41] identified that XAC2622 in *Xac* 306 may have the function of VirB7, although XAC2622 does not exhibit any sequence similarity with the VirB7 family. Thus, we re-annotated the *virB* genes in xanthomonads (Additional file 6), according to the relevant researches [36, 41].

Two genes, *traD* and *trwC*, encoding a coupling protein and a ralaxase respectively, were found to be adjacent to the *virB* operon (Additional file 2). The TraD protein, sitting at the inner membrane in contact with the assembled pilus and its scaffold as well as the relaxosome-plasmid DNA complex, is supposed to perform an essential coupling function in conjugative type IV secretion systems [42]. TrwC, the relaxase in the relaxosome, is a DNA strand transferase which functions during the conjugative cell to cell DNA transfer. It binds to the origin of transfer (*oriT*) and melts the double helix [43].

### Transposase and Tn*3* family transposon

**Fig. 4:**
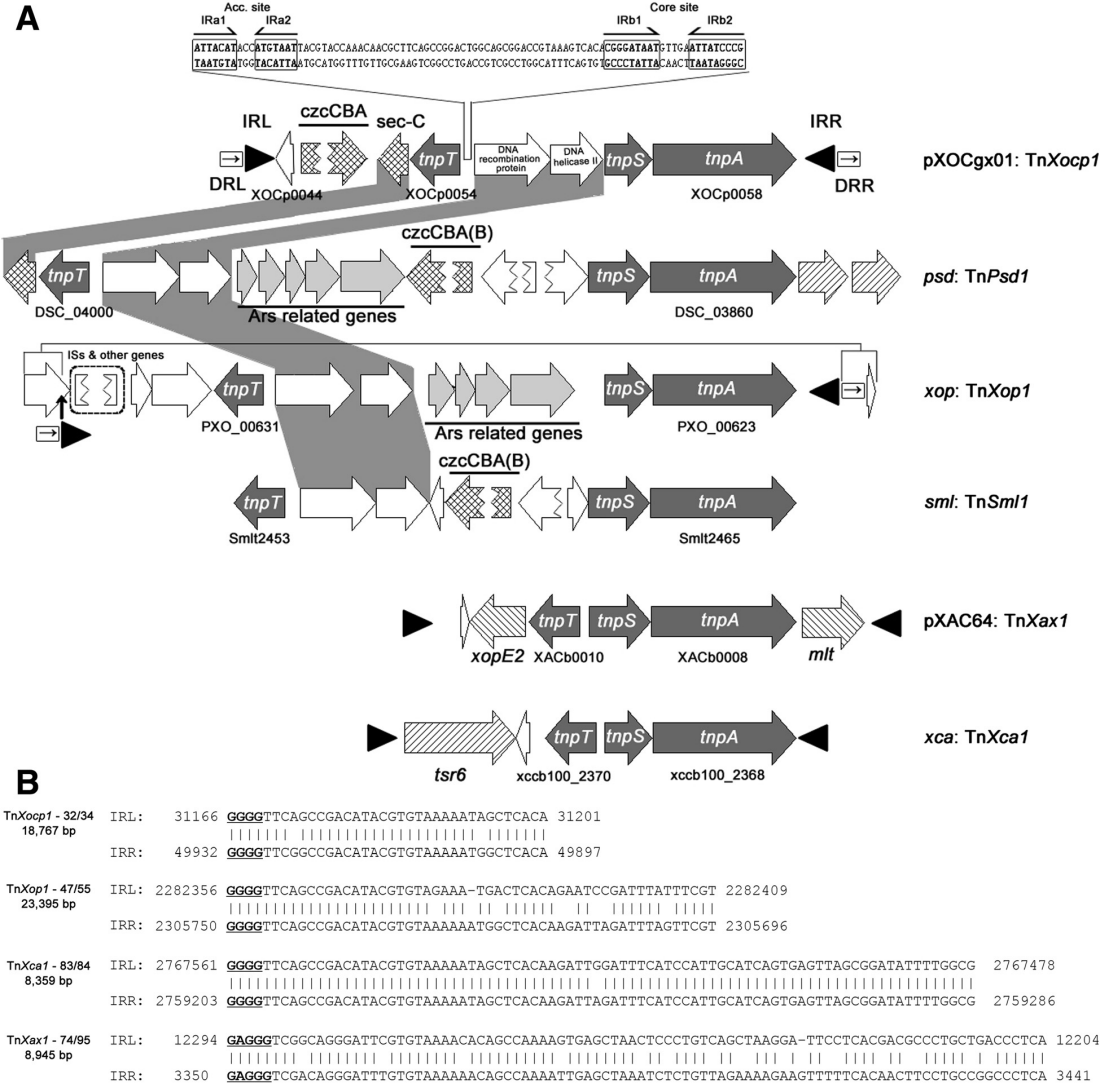
Genetic organizations of the Tn*Xocp1* cassette and IRs sequence analysis. **a** Genetic organization of the Tn*Xocp1* cassette located in plasmid pXOCgx01 and the alignment with Tn*Xocp1*-related structures in other genomes. Genes are indicated by different boxes with the direction of transcription shown by the arrowheads. Three core genes, *tnpA*, *tnpS* and *tnpT*, are shown in dark gray, and passenger genes related to czcCBA cluster in cross stripes, and genes related to arsenic resistance in pale gray, while other passenger genes in diagonal stripes or white boxes. The terminal inverted repeats (IRL and IRR) are shown as black triangles. The terminal direct repeats (DR) are shown as direct arrows in rectangle boxes. No IRs or DRs mean that there are no such sequences in those regions. Two segments with palindromes found in the *rst* region are supposed to be the Acc. site (17-bp IRa1 and IRa2 segment) for TnpT to bind and the core site (23-bp IRb1 and IRb2 segment) recognized by the TnpS recombinase. **b** IRs sequences identified from pXOCgx01 and other three *tnpA*-*tnpS*-*tnpT* cassettes. All of them are sharing high sequence similarities to Tn*Xocp1* IRs and begin with GGGG except Tn*Xax1*, which begins with GAGG. Abbreviations for genomes with *tnpA*-*tnpS*-*tnpT* cassette: *psd*, *Pseudoxanthomonas spadix* BD-a59; *xop*, *Xanthomonas oryzae* pv. *oryzae* PXO99^A^; *sml*, *Stenotrophomonas maltophilia* K279a; pXAC64, *Xanthomonas citri* pv. *citri* 306 plasmid pXAC64; *xca*, *Xanthomonas campestris* pv. *campestris* B100

pXOCgx01 harbors two ORFs encoding an imperfect transposase (XOCp0038) and a transposase (XOCp0058, TnpA) with DDE motif respectively. The region including the transposase gene (*tnpA*) and two recombination genes, namely *tnpS* and *tnpT*, was identified as a Tn*3*-like transposon [44], and was designated as Tn*Xocp1* in plasmid pXOCgx01. The genetic structure of *tnpA*-*tnpS-tnpT* can be found in both chromosomes and plasmids of some Gram-negative bacteria [44–46], in which *tnpA* and *tnpS* are closely linked. However, the gene composition between *tnpS* and *tnpT* is diverse in different bacteria (Fig. 4a), indicating a highly variable region.

Recently, Ferreira et al. found that Tn*3*-like transposons, e.g., Tn*Xax1*, might play a major role in the spread of pathogenicity determinants [46]. Tn*Xax1* found in plasmid pXAC64 of *Xac* 306 has a typical structure of Tn*3* family structure, and similar genetic organizations were widely distributed in *Xanthomonas* species [46]. At the left and right ends of the Tn*Xax1*-related structures, different T3SE genes like *xopE2*, *xopC* or TALE (**t**ranscription **a**ctivator-**l**ike **e**ffector) genes, and other passenger genes such as *mlt*, were found. The *tnpA*-*tnpS*-*tnpT* cassette is flanked by IRs (inverted repeats). Certain genes are flanked by the same IRs as that found in Tn*Xax1* to form mobile insertion cassettes (MICs). This may serve as a DNA shuttle vehicle, carrying passenger genes and transmitting horizontally among genomes. Obviously, there are no genes between *tnpS* and *tnpT* in Tn*Xax1* and Tn*Xax1*-related structures, which represent one type of Tn*3*-like transposon [46].

In pXOCgx01, we found that Tn*Xocp1* carries passengers not only at left end of *tnpT*, but also in the intergenic region between *tnpS* and *tnpT* where the *rst* (**r**esolution associated with Tnp**S** and Tnp**T**) region localizes [45]. The *tnpS* and *tnpT* promoters and two segments with palindromes, namely the Acc. site (17-bp IRa1 and IRa2 segments) for TnpT to bind and the core site (23-bp IRb1 and IRb2 segments) recognized by the TnpS recombinase were found in the functional *rst* region, similarly with the transposon Tn*4651* described by Yano [45]. Similar structures were also found in *X. oryzae* pv. *oryzae* PXO99^A^, *Stenotrophomonas maltophilia* K279a, and *Pseudoxanthomonas spadix* BD-a59 (Fig. 4a), and were designated as Tn*Xop1*, Tn*Sml1*, and Tn*Psd1* respectively. In addition to the similar structure of *tnpA*-*tnpS-tnpT*, Tn*Xocp1*, Tn*Xop1*, Tn*Sml1*, and Tn*Psd1* carried two other allelic homologous genes, encoding DNA recombination protein and DNA helicase II, respectively. Moreover, in both plasmid pXOCgx01 and *P. spadix* strain BD-a59, a gene encoding sec-C metal-binding protein next to *tnpT* was found, and no other similar structure was found in other genomes. Although the *czcCBA* locus also exists in Tn*Psd1* and Tn*Sml1*, it differs from the locus in Tn*Xocp1*. Genes related to arsenic resistance are in both Tn*Psd1* and Tn*Xop1* with a high similarity, but a 348-bp long ArsR family transcriptional regulator is found in Tn*Psd1* but not in Tn*Xop1*. The above information suggests that the Tn*Psd1* organization might represent one kind of ancestral and relatively intact transposons from which Tn*Xocp1*, Tn*Xop1* and Tn*Sml1* were formed by rearrangements.

Typical IR sequences were found in Tn*Xocp1*, Tn*Xop1*, Tn*Xax1* and Tn*Xca1*, but not in Tn*Psd1* and Tn*Sml1*. The DR (**d**irected **r**epeat) sequences found in Tn*Xocp1* and Tn*Xop1* may suggest they are new insertions relative to the others. With a length of 18,767 bp, at both flanks of Tn*Xocp1*, IR sequences were found as shown in Fig. 4b. They are 34 bp long with 32 bp identical, shorter than that of Tn*Xop1*, Tn*Xax1* and Tn*Xca1*. Both of Tn*Xocp1* and Tn*Xop1*, 5-bp DR sequences outside IRs were found, GGATA for Tn*Xocp1* and AAGGG for Tn*Xop1*. But no DR sequences were found flanking Tn*Xax1* and Tn*Xca1*, and no IR sequences in Tn*Psd1* and Tn*Sml1*. Not like the Tn*Xax1*-like structures carrying passenger genes involved in pathogenicity, the Tn*Xocp1*-like structures harbor heavy-metal resistance clusters, which may help the bacteria adapt to the adverse environment with heavy metal stresses.

The TnpA of Tn*Xocp1* shares 41.1 % protein sequence identity (with 98.9 % query coverage) with that of Tn*Xax1*, indicating that Tn*Xocp1* may belong to a new type of transposon. We submitted Tn*Xocp1* to the IS Finder database under the given name TnXo19. Genome analysis indicated that there are no Tn*3* family transposons in the genome (chromosome) of *Xoc* BLS256 and draft chromosome of GX01, but there are typical MIC structures in both chromosomes. Whether the Tn*Xocp1* can accelerate the transposition of the pathogenicity determinants in GX01 than those in BLS256 is noteworthy in future.

### pXOCgx01 is a conjugative plasmid

*Xoc* GX01 has been tested to be susceptible to all the commonly used antibiotics in our lab, such as kanamycin, ampicillin, spectinomycin, chloramphenicol, gentamycin and tetracycline. Moreover, no antibiotic resistance genes were found in plasmid pXOCgx01, so an insertional derivative of pXOCgx01, namely pXOCgx01::Tn5, was generated to place a kanamycin resistance marker but without mutating any pXOCgx01 genes. An R6Kγ conditional origin (R6Kγ*ori*) was introduced into pXOCgx01 with the insertion of Tn5, so the derivative of plasmid pXOCgx01::Tn5 could replicate in *E. coli* EC100D *pir*^+^, which has a *pir* gene to express the Π protein. In order to find out whether plasmid pXOCgx01 could replicate in *E. coli* and *Xanthomonas* spp. or not, pXOCgx01::Tn5 was introduced into *E. coli* DH5α, *Xoo* PXO99^A^, *Xcc* 8004 and one plasmidless *Xoc* isolate by electroporation, and clones were selected on solid medium with kanamycin. The result indicated that pXOCgx01 could replicate in *Xanthomonas* spp., but not in *E. coli* DH5α.

**Fig. 5:**
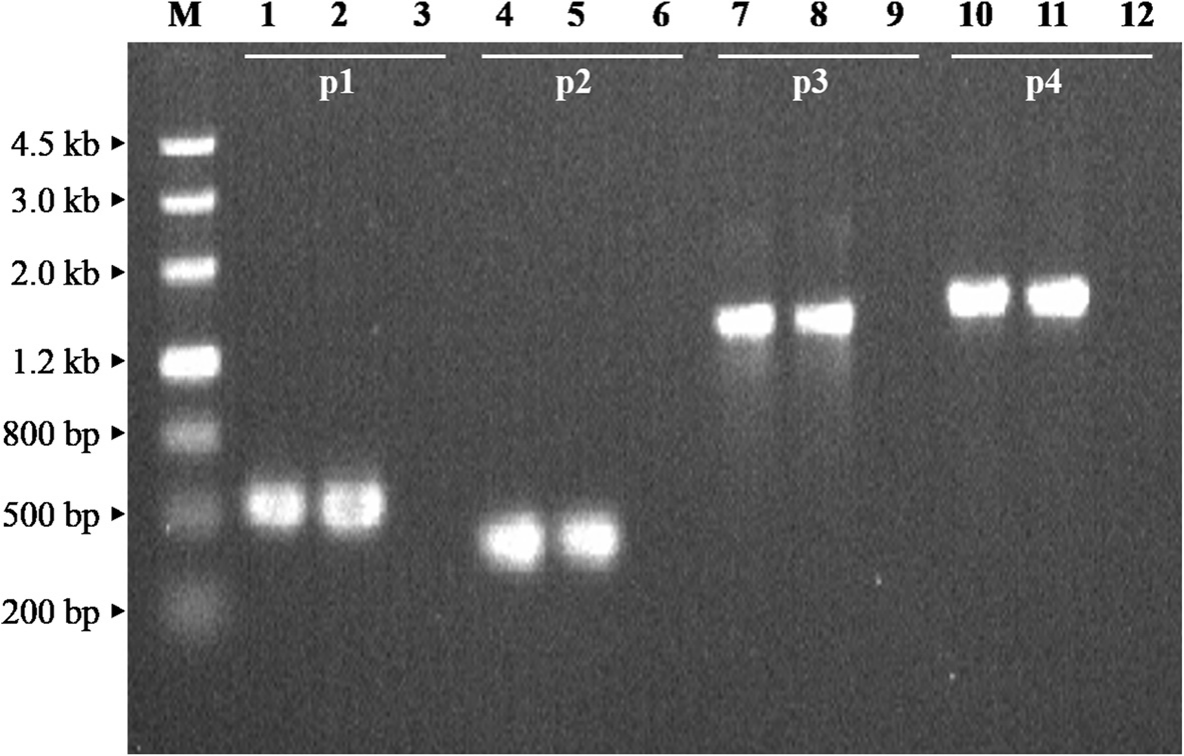
Evidence for conjugative transfer of pXOCgx01::Tn5 from EC100 *pir* ^+^ to *Xoo* PXO99^A^. PCR products electrophoresis analysis on 1.2 % agarose gel. M denotes marker III (TianGen). p1, 2, 3 and 4 denote products using primers Tn5-R6K-F/R, pXOC- tra -F/R, pXOC-virF/R and pXOC-res-F/R, respectively. Lane 1, 4, 7, 10, EC100 *pir* ^+^/pXOCgx01::Tn5 gDNA as a template; lane 2, 5, 8, 11, *Xoo* PXO99^A^/pXOC::Tn5 gDNA as a template; lane 3, 6, 9, 12, *Xoo* PXO99^A^ gDNA as a template

Since pXOCgx01 appeared to carry *virB* locus encoding a T4SS, the transmissibility of the plasmid was investigated. EC100D *pir*^+^/pXOCgx01::Tn5 was used as a donor to track conjugal transfer of pXOCgx01 to *Xoo* PXO99^A^. Plasmid DNA was isolated from the putative transconjugants by the modified alkaline lysis method and digested with *Bam*HI. Fragment profiles were examined with agarose gel electrophoresis. Plasmid profiles of the transconjugants were the same as that of EC100D *pir*^+^/pXOCgx01::Tn5 (data not shown). The pXOCgx01-specific PCR assay was done, and the evidence is shown as Fig. 5. From the plasmid profiles and PCR assay results, pXOCgx01 was experimentally determined to be self-transferable by conjugation, without transfer helper strains, from *E. coli* EC100D *pir*^+^ to *Xoo* PXO99^A^, indicating that pXOCgx01 is a conjugative plasmid.

### pXOCgx01 enhanced the heavy metal tolerance of *Xoo* PXO99^A^

**Fig. 6:**
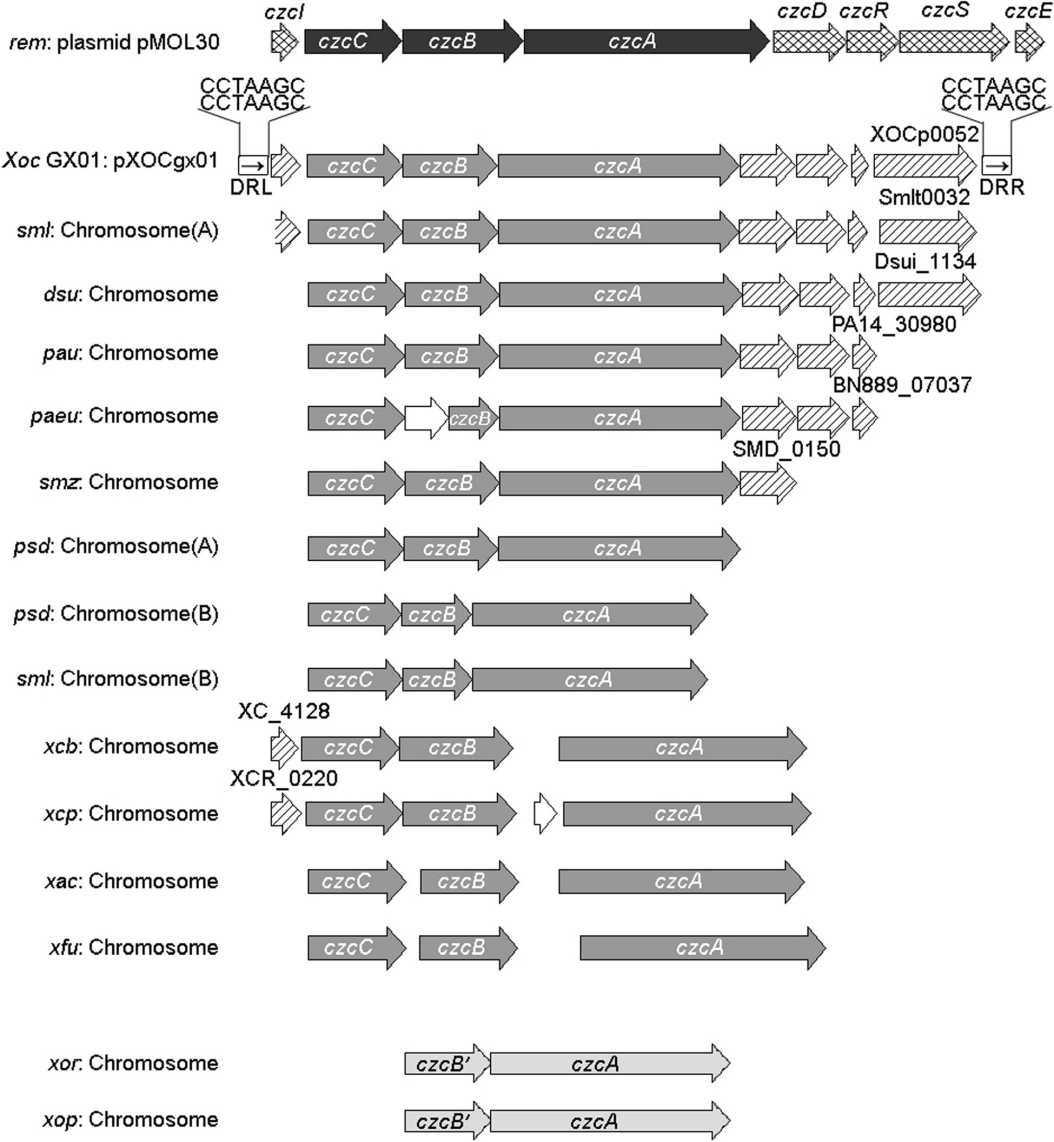
Genetic structure of the *czcCBA* system located in plasmid pXOCgx01 and alignment with other genomes. The core genes, *czcA*, *czcB* and *czcC*, are shown in gray. Genes of *czcCBA* cluster on plasmid pMOL30 were filled with different markers from pXOCgx01 denoting that those genes have a low similarity with pXOCgx01. Direct arrows in rectangle boxes at the flanking regions of *czcCBA* cluster on pXOCgx01 denote direct repeat sequences

The *czcCBA* locus on pXOCgx01 contains three Cobalt-zinc-cadmium resistance protein genes *czcA*, *czcB*, and *czcC*, the products of which constitute a membrane-bound protein complex catalyzing an energy-dependent efflux of the three metal cations, Co^2+^, Zn^2+^, and Cd^2+^. The archetype CzcCBA, an RND (**r**esistance-**n**odulation-**d**ivision) system for HME (**h**eavy **m**etal **e**fflux), was first reported in plasmid pMOL30 of *Cupriavidus metallidurans* CH34, consisting of *czcCBA* and flanking regulatory genes [47]. Phylogenetic analysis showed that the *czcCBA* locus on pXOCgx01 shared a low sequence similarity against that in pMOL30, but high similarities to those in chromosomes of *Stenotrophomonas maltophilia* K279a, *Dechlorosoma suillum* PS, and *Pseudomonas aeruginosa* PA38182. It is noteworthy that there is one pair of direct repeat sequence at both ends of the pXOCgx01 *czcCBA* locus, indicating a recent horizontal gene transfer event. The BLAST results showed that the core *czcCBA* genes are conserved, but their flanking genes are variable in many other bacterial strains, in some cases only *czcCBA* or *czcBA* genes remaining (Fig. 6). It is also worth noting that the archetype pMOL30 *czc* or pXOCgx01 *czcCBA* ortholog genes are absent from the sequenced genomes of *Xoo* strains and *Xoc* BLS256, in which only a *czcA* gene was annotated, suggesting a series of genes trimming or rinsing during the evolution of *czcCBA* loci.

**Fig. 7:**
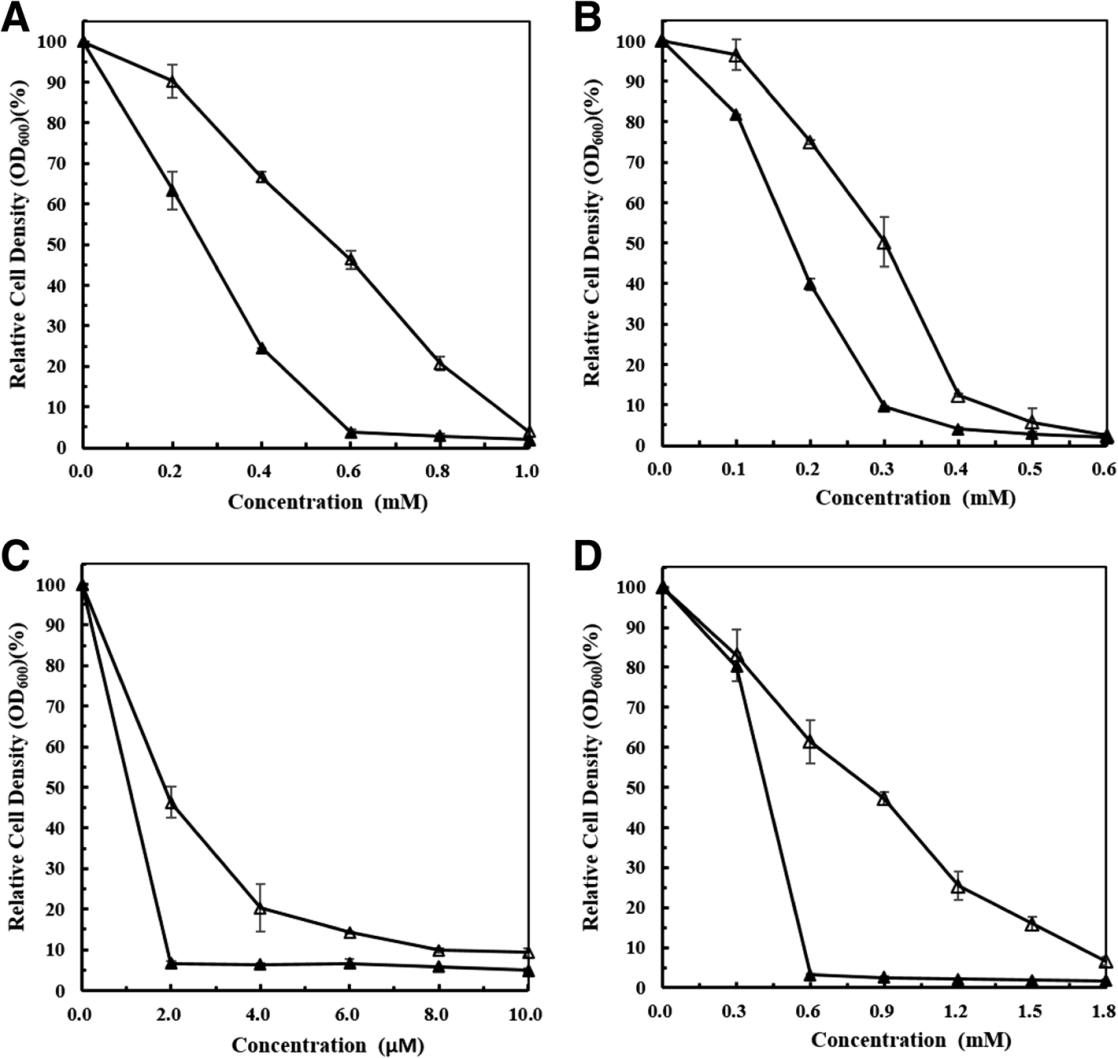
Co^2+^, Zn^2+^, Cd^2+^, Ni^2+^ tolerance analysis of *Xoo* PXO99^A^ and the derivative strain in NB medium. ▲, wild-type strain *Xoo* PXO99^A^; ∆, strain *Xoo* PXO99^A^ harboring pXOCgx01::Tn5. **a** CoCl_2_ tolerance analysis; **b** ZnSO_4_ tolerance analysis; **c** CdSO_4_ tolerance analysis; **d** NiCl_2_ tolerance analysis. Strains were inoculated into 10 mL NB medium with a final concentration of OD_600_ = 0.01, and incubated at 28 °C with shaking at 200 rpm. Bacterial growth was determined by measuring OD_600_ 22 h after inoculation. Relative cell density means the percentage of each strain’s cell density at different heavy metal concentrations versus the cell density when no heavy metal added. Data presented are from a representative experiment; similar results were obtained in other two independent experiments

To determine the function of pXOCgx01, we tested the strain *Xoo* PXO99^A^/pXOCgx01::Tn5 under the stresses of heavy metals and other chemicals. The results showed that the introduction of pXOCgx01::Tn5 into *Xoo* PXO99^A^ could significantly enhance tolerances to Co^2+^, Zn^2+^, Cd^2+^ and Ni^2+^ (Fig. 7), but did not influence tolerances to Cu^2+^, Mn^2+^, Fe^3+^, organic phenol, SDS or H_2_O_2_, and showed no contribution to the adaptability under high osmotic pressure or different pH levels. Besides, the cell growth curve analysis showed no significant differences between *Xoo* PXO99^A^ and the derivative harboring plasmid pXOCgx01 (data not shown). These plasmid-related phenotypes, namely tolerances to Co^2+^, Zn^2+^, Cd^2+^ and Ni^2+^, demonstrated the function of *czcCBA* locus. The rice field from which *Xoc* GX01 was isolated is near the Southern China lead-zinc mine districts and the background value of heavy metals is relatively high [48, 49]. The cadmium content in the arable layer (20 cm) of the reclaimed farmland near by the site where GX01 was isolated is 14.93 mg/kg in average (Gui-Rong Wu, personal communication). Thus, it is rational to explain that *Xoc* GX01 harboring the *czcCBA* locus on pXOCgx01.

### Curing of plasmid pXOCgx01 from *Xoc* GX01

To get a pXOCgx01-free derivative of strain GX01, all kinds of attempts, including elevated temperatures, the SDS-elevated temperature-crossed method, acridine orange stress tests, electrocompetent cells electric pulse tests, plasmid incompatibility tests (pLAFR6, pPH1JI, pBBad18K, pUFR034, pBBR1MSC-5), were adopted to cure plasmid pXOCgx01. By PCR assay and plasmid-isolation detection, no pXOCgx01-free derivatives were gotten. The putative origin linked with the EZ-Tn5™ < R6Kγori/KAN-2 > transposon was introduced into the wild-type strain GX01 by electroporation, but interestingly, the recombinant plasmid could coexist with pXOCgx01 in strain GX01 and showed no incompatibility. The failure to cure plasmids from their hosts is also found in *X. arboricola* pv. *pruni* CFBP 5530 harboring plasmid pXap41 [12]. The results may indicate a stability relationship between GX01 and the recalcitrant plasmid pXOCgx01, not just for surviving, but also for advantages in environmental adaptations.

### Survey of plasmids in *Xoc* Chinese isolates

To examine the distribution of indigenous plasmids in *Xoc* Chinese isolates, we tested up to 257 *Xoc* strains isolated from Anhui (*n* = 79), Fujian (*n* = 13), Guangxi (*n* = 75), Guangdong (*n* = 22), Hainan (*n* = 58), Jiangsu (*n* = 1), Yunnan (*n* = 1) and Zhejiang (*n* = 8) provinces, using both the conventional plasmid isolation method and the kit method. Isolated plasmid DNA samples were digested with different restriction endonucleases and followed by agarose gel electrophoresis detections. We found that nine different isolates, of which seven isolated from Guangxi Province, one from Guangdong Province and one from Hainan Province, harbor at least one plasmid in each strain according to their plasmid profiles. The plasmid survey suggested that the presence of pXOCgx01 in *Xoc* GX01 was not an individual event and it is worthwhile studying more about plasmid functions in *Xoc*.

## Discussion

Although plasmids have been considered to be widespread in bacteria, there is no any report about plasmids in *Xoc* strains. In this paper we reported an indigenous plasmid pXOCgx01 from a *Xoc* strain isolated from the central area of South China rice growing region, one of the putative cradles of rice *Oryza sativa* [26]. The plasmid, comprising six regions with distinct origins, has a chimeric structure, indicating that the generation of pXOCgx01 might be a result of multiple DNA rearrangement events. pXOCgx01 has been demonstrated to be a conjugative plasmid, displaying the biological function of the *virB* locus. The introduction of pXOCgx01 to *Xoo* PXO99^A^ did significantly promote tolerances to certain heavy metals, but did not enhance the virulence. A plasmid survey indicated that at least 9 different plasmids exist in our *Xoc* Chinese isolates. As a model system for studying *Xoc-*rice interaction [1], it is of importance to assess the impact of indigenous plasmids of these phytopathogenic bacteria on metabolism, persistence and pathogenicity.

The *virB* locus in pXOCgx01 encodes a type IVA system (T4ASS). It has been proposed that T4SSs were evolved from preexisting sources, first into conjugation systems, and later into virulence systems [50]. Our results indicated that the pXOCgx01 T4SS might mediate the plasmid transferring, but not virulence. Similar results also obtained in other *Xanthomonas* species. El Yacoubi et al. [17] presented evidence that *in planta* the transfer of a 37 kb plasmid (pXcB) from *X. aurantifolii* to *X. citri* could occur via T4SS. In *Xcc* 8004, the T4SS deletion mutant displayed the same virulence as the wild type strain [51]. Recent studies showed that the T4SS is not induced during the infection process in *X. citri*, and is not involved in infection process in citrus, but may be very important in cell-to-cell communication [52]. These results at least suggested that T4SS is not the determinant of pathogenicity in some xanthomonads, unlike the T4SS in *A. tumefaciens*, *Helicobacter pylori*, and *Legionella pneumophila* [53].

Plasmid pXOCgx01 has a mosaic structure both on gene context and gene contents, showing a high recombination and heterogeneity. Transposases or recombinases catalyze DNA transpositions, which are involved in DNA rearrangements and horizontal gene transfers. pXOCgx01 harbors a *tnpA*-*tnpS*-*tnpT* locus which was found to be widely distributed in prokaryotic world, such as the important lab strain *Xoo* PXO99^A^, but absent from the American strains [54]. In some pathogenic bacteria, this kind of loci was found to be closely linked to virulence genes and avirulence genes, suggesting the pathogenic potential of Tn*5044* [44]. Unlike Tn*Xax1* in pXAC64 [46], Tn*Xocp1* in plasmid pXOCgx01 carries no pathogenicity genes but genes involved in heavy metal tolerance.

Transition metals are essential micronutrients for healthy immune function for all living organisms, but a high level of these metals will potentiate toxicity to organisms, so transition metal ion homeostasis must be carefully regulated. To compete for limited metals and simultaneously to prevent metal toxicity within the host, pathogens have developed a series of metal regulatory, acquisition, and efflux systems [55]. Plasmid-mediated detoxification or resistance is always one of the major concerns in modern microbiology. Although plasmid pXOCgx01 were susceptible to the commonly used antibiotics tested in our lab, the introduction of pXOCgx01 into *Xoo* PXO99^A^ could significantly increase the tolerance to heavy metals, indicating the function of *czcCBA* genes. The high heavy metal concentration of the region, from where *Xoc* GX01 was isolated, may provide a communal gene pool for strains [56] to adopt a CzcCBA-encoding plasmid for selective advantages.

## Conclusions

In this study, we reported the identification and analysis of an indigenous plasmid, pXOCgx01, from *Xoc* GX01. pXOCgx01 is the first indigenous plasmid from *Xoc*, and the first completely sequenced plasmid from *Xanthomonas oryzae* species. pXOCgx01 has been demonstrated being a conjugative plasmid and can significantly enhance the tolerance of *Xoo* PXO99^A^ to heavy metals. The *virB* genes, *czc* genes and the mobile insertion cassette on the pXOCgx01 are absent from other sequenced *Xanthomonas oryzae* strains. These results may be helpful for elucidation of adaptive evolution of *Xoc*.

## Additional files

Additional file 1: Table S1. Putative ORFs of pXOCgx01. (XLSX 22 kb) Additional file 2: Figure S1. The phylogenetic tree analysis of the whole sequence of plasmid pXOCgx01 with other plasmids. (JPEG 2166 kb) Additional file 3: Figure S2. The BLASTN alignment of plasmid pXOCgx01 with other whole plasmid and genome sequences. (TIFF 3082 kb) Additional file 4: Table S2. Specific locations in corresponding genomes used in the phylogenetic tree. (DOCX 18 kb) Additional file 5: Table S3. The comparison of type IVA genes of *Xanthomonas* with whole genome sequenced. (DOCX 19 kb) Additional file 6: Table S4. Primers used in this study. (DOCX 14 kb)

## Abbreviations

*Xoc*: *Xanthomonas oryzae* pathovar *oryzicola*
*Xoo*: *Xanthomonas oryzae* pathovar *oryzae*
*Xcc*: *Xanthomonas campestris* pathovars *campetris*
BLS: Bacterial leaf streak
ORFs: Open reading frames
IRs: Inverted repeats
*rst*: Resolution associated with TnpS and TnpT
DR: Directed repeat
RND: Resistance-nodulation-division
HME: Heavy metal efflux
COG: Clusters of orthologous groups
CDD: Conserved domain database
TALEs: Transcription activator-like effectors
