# Supplementary figures and images for: Complete sequence and detailed analysis of the first indigenous plasmid from Xanthomonas oryzae pv. oryzicola
Source: BMC Microbiol. 2015 Oct 24;15:233. doi: 10.1186/s12866-015-0562-x (PMC4619425; doi:10.1186/s12866-015-0562-x)

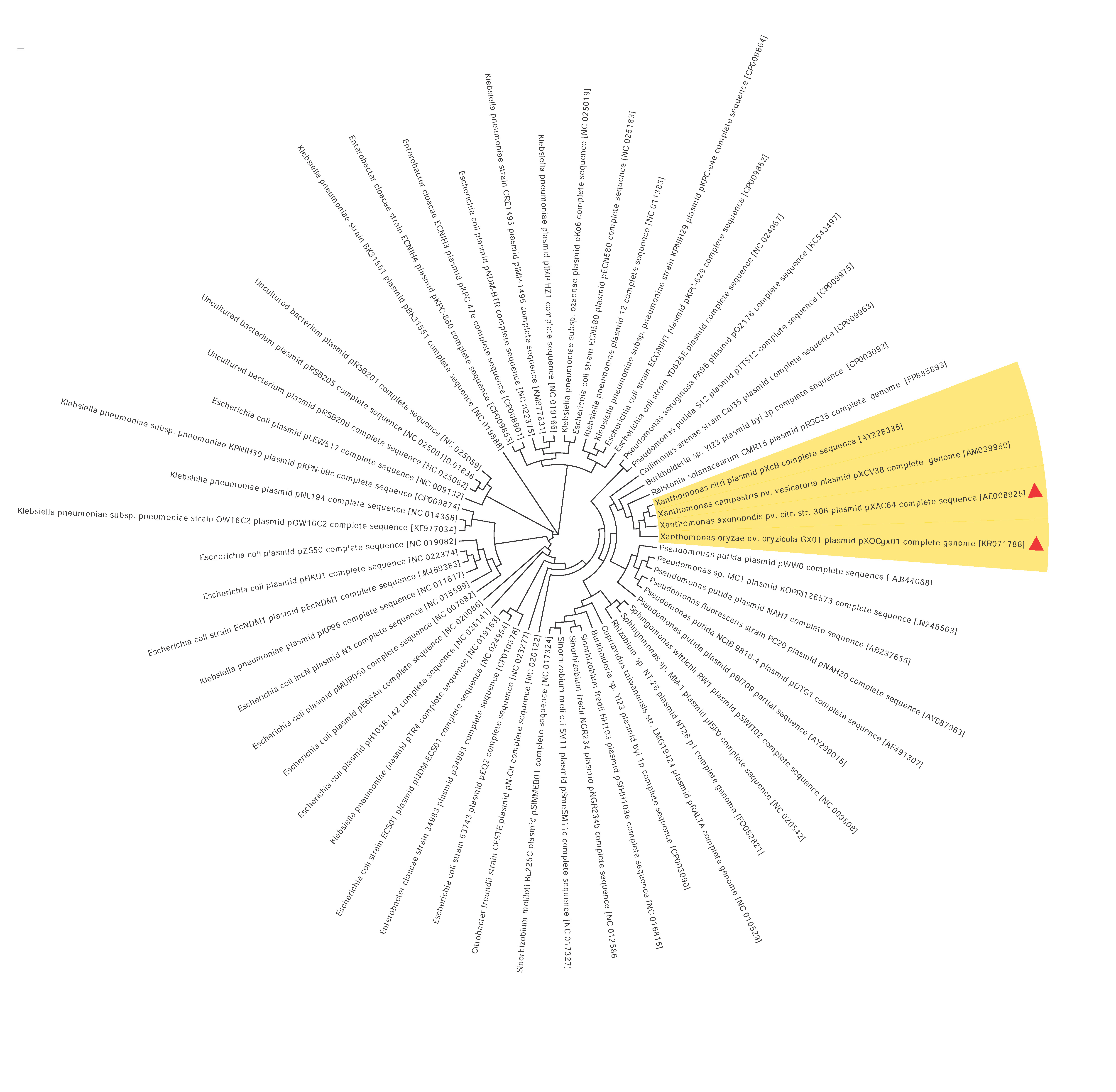

Supplement: Additional file 2: Figure S1. — The phylogenetic tree analysis of the whole sequence of plasmid pXOCgx01 with other plasmids. (JPEG 2166 kb) [file 12866_2015_562_MOESM2_ESM.jpg]

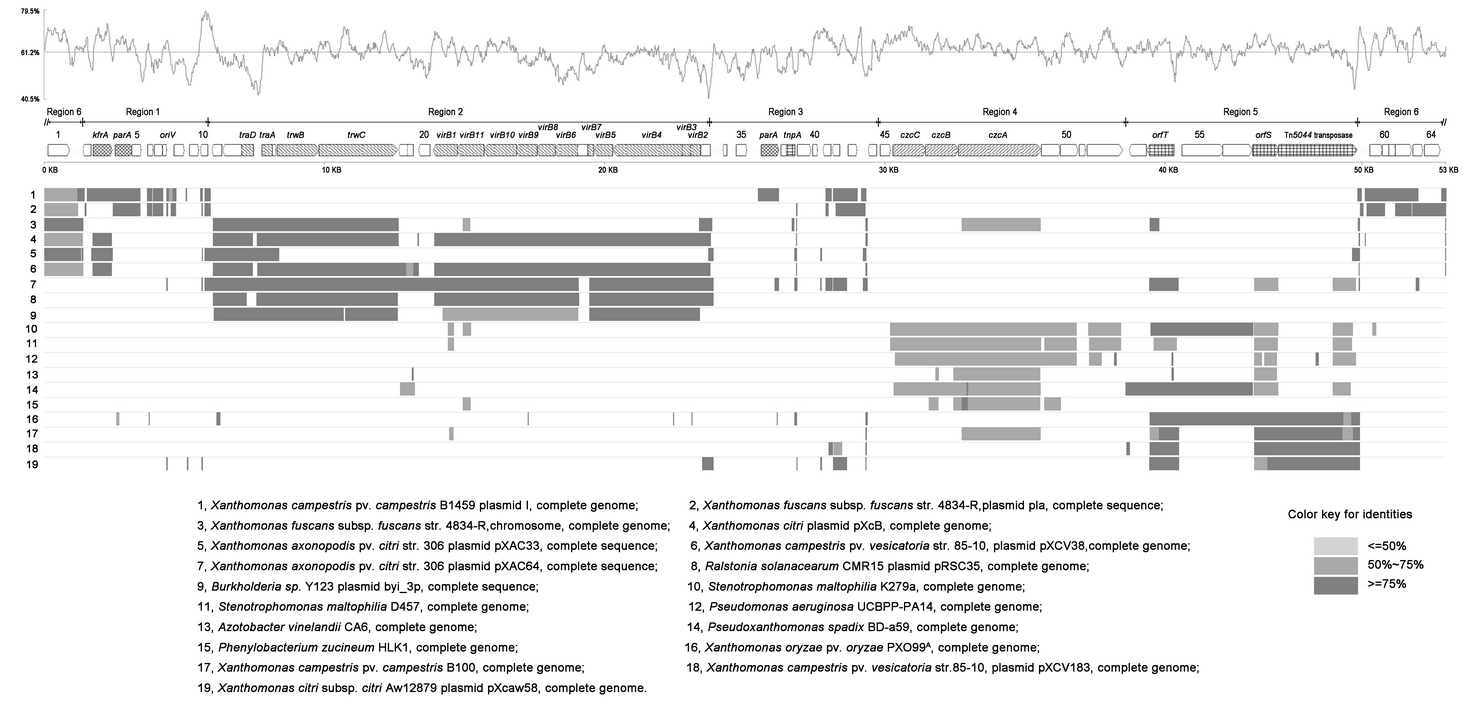

Supplement: Additional file 3: Figure S2. — The BLASTN alignment of plasmid pXOCgx01 with other whole plasmid and genome sequences. (TIFF 3082 kb) [file 12866_2015_562_MOESM3_ESM.tif]
