# Supplementary material for: Complete sequence and detailed analysis of the first indigenous plasmid from Xanthomonas oryzae pv. oryzicola
Source: BMC Microbiol. 2015 Oct 24;15:233. doi: 10.1186/s12866-015-0562-x (PMC4619425; doi:10.1186/s12866-015-0562-x)
Supplement: Additional file 4: Table S2. — Specific locations in corresponding genomes used in the phylogenetic tree. (DOCX 18 kb) [file 12866_2015_562_MOESM4_ESM.docx]

Additional file 4: Table S2 Specific locations in corresponding genomes used in the phylogenetic tree

| Sequence locus | Genome | Positions |
| --- | --- | --- |
| Region 1： krfA locus | pXOCgx01 | 1,641 to 4,105 |
|  | *Xcc* B1459 plasmid : I | 3,226 to 5,262 joined with 5,587 to 5,791 |
|  | *xfu* plasmid pla | 34,592 to 34,774 joined with 35,056 to 36,080 |
|  | *xac* plasmid pXAC33 | 32,816 to 33,624 |
|  | *xcv* plasmid pXCV38 | 4,882 to 5,601 |
|  | *X. citri* plamid pXcB | 25,218 to 25,937 |
| Region 2: T4SS locus | pXOCgx01 | 6,403 to 25,393 |
|  | *xac* plasmid pXAC64 | 22,854 to 24,971 joined with 26,603 to 32,070 and 32,447 to 38,542 and 39,225 to 43,573 |
|  | *byi* plasmid byi_3p | 87,735 to 91,642 joined with 92,367 to 95,944 and 97,025 to 97,512 and 101,020 to 103,558 and 104,833 to 105,651 |
|  | *rsm* plasmid pRSC35 | 7,462 to 8,282 joined with 8,720 to 8,835 and 8,999 to 12,072 and 12,171 to 14,178 and 20,301 to 24,773 and 26,364 to 30,675 |
|  | *X. citri* plamid pXcB | 2,820 to 7,138 joined with 7,853 to 11,427 and 12,379 to 13,313 and 15,721 to 21,123 and 21,335 to 22,427 |
|  | *xcv* plasmid pXCV38 | 9,182 to 9,249 joined with 10,444 to 11,475 and 11,711 to 12,332 and 14,339 to 19,837 and 21,982 to 22,916 and 23,871 to 27, 451 and 28,158 to 28,718 |
|  | *Pandoraea vervacti strain NS15 sp. nov plasmid* | 52,489 to 56,441 joined with 57,205 to 58,730 joined with 59,606 to 61,890 joined with 64,206 to 68,644 joined with 93,774 to 94,120 |
|  | *xfu* | 4,928,281 to 4,930,573 joined with 4,932,582 to 4,937,244 joined with 4,951,368 to 4,951,715 |
| Region 3: par locus | pXOCgx01 | 25,314 to 31,611 |
|  | *Xcc* B1459, plasmid I | 34,359 to 35,163 |
|  | *xfu* plasmid plc | 16,026 to 16,819 |
|  | *X.axonopodis* pv. *glycines* strain AG1 plasmid pAG1 | 11,681 to 12,412 |
|  | *X. arboricola* pv. *pruni str.* CF 5530 plasmid pXap41 | 11,846 to 12,261 |
| Region 4: czcABC locus | pXOCgx01 | 31,762 to 40,947 |
|  | Anisakis simplex genome assembly A_simplex ,scaffold ASIM_scaffold0000498 | 20,767 to 25,553 |
|  | *avd* | 810,921 to 813,300 |
|  | *pzu* | 1,636,935 to 1,640,264 |
|  | *pau* | 2,693,934 to 2,698,156 |
|  | *psd* | 1,672,142 to 1,675,615 |
|  | *smz* | 184,075 to 188,810 |
|  | *sml* | 33,656 to 39,222 |
|  | *rme* plasmid pMOL30 | 73,502 to 85,845 |
|  | *xop* | 2,219,413 to 2,223,613 |
|  | *xor* | 2,499,509 to 2,503,709 |
| Region 5: Tn*5044* lucos | pXOCgx01 | 40,948 to 49,932 |
|  | *xop* | 2,293,951 to 2,297,629 joined with 2,301,705 to 2,305,750 |
|  | *psd* | 663,937 to 668,724 |
|  | *xca* | 2,759,203 to 2,764,471 |
|  | *xcv* plasmid pXCV183 | 102,994 to 108,202 |
|  | *xci* plasmid pXcaw58 | 2,784 to 3,840 joined with 5,691 to 9,213 |
|  | *sml* | 2,479,288 to 2,482,363 |
|  | *xac* plasmid pXAC64 | 9,618 to 10,697 |
|  | *xcv* | 2,604,077 to 2,604,766 joined with 2,608,233 to 2,609,270 |
| Region 6：Hypothetical proteins locus | pXOCgx01 | 50,184 to 786 |
|  | *xac* plasmid pXAC33 | 635 to 1,461 |
|  | *Xcc* B1459 plasmid I | 43,552 to 45,534 joined with 881 to 1,873 |
|  | *xfu* plasmid pla | 37,198 to 39,312 joined with 39,741 to 40,015 and 115 to 1,167 |
|  | *xac* plasmid pXAC33 | 635 to 1,461 |
|  | *xcv* plasmid pXCV38 | 1,054 to 1,883 |
|  | *X. albilineans* str. GPE PC73, plasmid I | 21,196 to 21,905 |
|  | *X. citri* plamid pXcB | 883 to 1,715 |
