# Supplementary material for: Complete sequence and detailed analysis of the first indigenous plasmid from Xanthomonas oryzae pv. oryzicola
Source: BMC Microbiol. 2015 Oct 24;15:233. doi: 10.1186/s12866-015-0562-x (PMC4619425; doi:10.1186/s12866-015-0562-x)
Supplement: Additional file 5: Table S3. — The comparison of type IVA genes of Xanthomonas with whole genome sequenced. (DOCX 19 kb) [file 12866_2015_562_MOESM5_ESM.docx]

Additional file 5: Table S3 Comparison of type IVA genes of Xanthomonas with whole genome sequenced

| **Organism** | **Replicon** | ***virB1*** | ***virB2*** | ***virB3*** | ***virB4*** | ***virB5*** | ***virB6*** | ***virB7*** | ***virB8*** | ***virB9*** | ***virB10*** | ***virB11*** | ***virD4*** | **Reference*** |
| --- | --- | --- | --- | --- | --- | --- | --- | --- | --- | --- | --- | --- | --- | --- |
| *Xoc* GX01 | Chromosome | - | - | - | - | - | - | - | - | - | - | - | - | Our Unpublished |
|  | pXOCgx01 | + | + | + | + | + | + | (+) | + | + | + | + | <+> | KR071788.1 |
| *Xcv* 85-10 (*xcv*) | Chromosome | - | - | - | - | +2 | +4 | + | + | + | - | - | + | AM039952.1 |
|  | pXCV38 | + | + | - | + | + | + | (+) | + | + | + | + | <+> | AM039950.1 |
| *Xac* 306 (*xac*) | Chromosome | + | + | + | + | + | +3 | + | + | + | + | + | + | AE008923.1 |
|  | pXAC64 | + | + | + | + | + | + | (+) | + | + | + | + | <+> | AE008925.1 |
| *Xcc* Aw12879 (*xci*) | Chromosome | + | + | + | + | + | +3 | + | + | + | +2 | + | + | CP003778.1 |
|  | pXcaw19 | - | - | - | - | - | - | - | - | + | - | - | + | CP003779.1 |
| *Xac 29*-1 (*xao*) | Chromosome | + | + | + | +2 | - | +2 | + | + | + | - | - | + | CP004399.1 |
|  | pXAC47 | - | + | + | + | + | + | + | + | + | - | - | + | CP004401.1 |
|  | pXAC64# | + | + | + | + | + | + | - | + | + | + | 2+ | <+> | CP004400.1 |
| *X.* *albilineans* GPE PC73 (*xal*) | Chromosome | + | + | + | + | + | +4 | + | + | + | + | + | +6 | FP565176.1 |
|  | plasmI | + | - | + | + | + | + | - | + | + | + | + | - | FP340279.1 |
|  | plasmII | + | - | - | - | + | + | - | + | + | + | - | - | FP340278.1 |
|  | plasmIII | - | - | + | + | + | + | + | + | +- | + | + | + | FP340277.1 |
| *Xff* 4834-R (*xfu*) | Chromosome | + | + | + | + | + | + | + | + | + | - | + | + | FO681494.1 |
|  | pla | - | - | - | - | - | - | - | - | - | - | - | + | FO681495.1 |
|  | plb | - | - | - | - | + | + | - | - | - | - | - | - | FO681496.1 |
| *X. citri* | pXcB | + | + | + | + | + | + | + | + | + | + | + | <+> | AY228335.1 |
| *Xcc* B1459 | plasmidI | - | - | - | - | - | - | - | - | - | - | - | - | LN811400.1 |
| *Xcc* ATCC 33913(*xcc*) | Chromosome | + | + | + | + | + | +4 | + | + | + | + | + | +2 | AE008922.1 |
| *Xcc* 8004 (*xcb*) | Chromosome | + | + | + | +2 | +2 | +4 | + | + | + | + | + | +3 | CP000050.1 |
| *Xcc* B100 (*xca*) | Chromosome | + | + | + | +3 | + | +2 | + | + | + | + | + | + | AM920689.1 |
| *Xcr* 756C (*xcp*) | Chromosome | + | + | + | + | - | +7 | + | + | + | + | + | +3 | CP002789.1 |
| *Xoo* KACC 10331 (*xoo*) | Chromosome | - | - | - | - | + | + | - | - | - | - | - | - | AE013598.1 |
| *Xoo* MAFF311018 (*xom*) | Chromosome | - | - | - | - | - | - | - | - | - | - | - | - | AP008229.1 |
| *Xoo* PXO99A (*xop*) | Chromosome | - | - | - | - | - | - | - | - | - | - | - | - | CP000967.1 |
| *Xoc* BLS256 (*xor*) | Chromosome | - | - | - | - | - | - | - | - | - | - | - | - | CP003057.1 |
| *Xa* *citrumelo* F1 (*xax*) | Chromosome | - | - | - | - | - | - | - | - | + | - | + | +2 | CP002914.1 |
| *X. sacchari* R1 (*xsa*) | Chromosome | - | - | - | - | - | - | - | - | + | - | + | - | CP010409.1 |
| *S. maltophilia* K279a (*sml*) | Chromosome | + | +2 | +2 | +2 | + | + | + | + | +2 | + | +2 | +2 | AM743169.1 |
| *S. maltophilia* R551-3 (*smt*) | Chromosome | + | + | + | + | - | + | + | + | + | + | + | + | CP001111.1 |
| *S. maltophilia* JV3 (*buj*) | Chromosome | + | + | + | + | - | + | + | + | + | + | + | + | CP002986.1 |
| *S. maltophilia* D457 (*smz*) | Chromosome | + | + | + | +2 | - | + | + | + | + | - | +3 | +2 | HE798556.1 |

Note: “+” possessing gene, “-” no gene, “(+)” putative *virB7* [1], <+> putative TraG/TraD family gene. The numerical figure indicates the copy numbers of the certain gene. * All references are GenBank Accession Numbers.

**References**

[1] Alegria MC, Souza DP, Andrade MO, Docena C, Khater L, Ramos CHI, et al. Identification of new protein-protein interactions involving the products of the chromosome- and plasmid-encoded type IV secretion loci of the phytopathogen *Xanthomonas axonopodis* pv. *citri*. J Bacteriol 2005; 187(7): 2315-25.
