# Supplementary material for: Complete sequence and detailed analysis of the first indigenous plasmid from Xanthomonas oryzae pv. oryzicola
Source: BMC Microbiol. 2015 Oct 24;15:233. doi: 10.1186/s12866-015-0562-x (PMC4619425; doi:10.1186/s12866-015-0562-x)
Supplement: Additional file 6: Table S4. — Primers used in this study. (DOCX 14 kb) [file 12866_2015_562_MOESM6_ESM.docx]

**Additional file 6: Table S4 Primers used in this study.**

| Primer pairs | Target Locus | Sequence (5’ to 3’) | Product Size (bp) |
| --- | --- | --- | --- |
| Plasmid pXOCgx01 | | | |
| pXOC-virF/R | XOCp0022 and  XOCp0023 | ACGATTTGAACGCGGTAG/  TGGTGGCCGATGATCGCAAG | 1,463 |
| pXOC-res-F/R | XOCp0036 to  XOCp0039 | GATGGCACCGTGAATGGTC/  ACAGCCAGCACCAAGCCAAG | 1,594 |
| pXOC-tra-F/R | XOCp0058 | ATGGCCAAGTGGTCATCGGC/  AGGCCGGCGTAGCTGTTGAT | 394 |
| Primers to establish Tn5 cassette in pXOCgx01::Tn5 | | | |
| Tn5-R6K-F/R | -- | ACGAAACACGGAAACCGAAGAC/  AACATCATTGGCAACGCTACCT | 578 |
| Primers to localize Tn5 insertion site in pXOCgx01::Tn5 | | | |
| Tn5KAN2-F/R | -- | CCTACAACAAAGCTCTCATCAACC/  CTACCCTGTGGAACACCTACATCT | -- |
